# Subdiaphragmatic vagotomy promotes tumor growth and reduces survival via TNFα in a murine pancreatic cancer model

**DOI:** 10.18632/oncotarget.15019

**Published:** 2017-02-02

**Authors:** Lars Ivo Partecke, André Käding, Dung Nguyen Trung, Stephan Diedrich, Matthias Sendler, Frank Weiss, Jens-Peter Kühn, Julia Mayerle, Katharina Beyer, Wolfram von Bernstorff, Claus-Dieter Heidecke, Wolfram Keßler

**Affiliations:** ^1^ Department of General, Visceral, Thoracic and Vascular Surgery, University Medicine, Greifswald, Germany; ^2^ Department of Internal Medicine A, University Medicine, Greifswald, Germany; ^3^ Department of Experimental Radiology, University Medicine, Greifswald, Germany; ^4^ Current address: Department of General, Visceral and Vascular Surgery, Charité–University Medicine, Campus Benjamin Franklin, Berlin, Germany

**Keywords:** vagus nerve, TNFα, pancreatic cancer, macrophages, murine cancer model

## Abstract

This study analyses the effects of vagotomy on tumor growth and survival in a murine, pancreatic cancer model in wild-type and TNFα-knockout (−/−) mice.

Throughout many operative procedures in the upper gastrointestinal tract the partial or complete transection of the vagus nerve or its local nerve fibers is unavoidable. Thereby its anti-inflammatory effects in residual tumor tissue may get lost. This effect may be mediated by tumor-associated macrophages (TAM) secreting TNFα.

In an orthotopic murine pancreatic cancer model subdiaphragmatic vagotomy *versus* sham surgery was performed. The impact on tumor growth was monitored in wild type and TNFα −/− mice using MRI. TAMs as well as expression levels of TNFα were analyzed using immunohistochemistry. The role of TNFα on tumor growth and migration was examined *in vitro*. Vagotomised mice showed increased tumor growth with macroscopic features of invasive growth and had a shorter survival time. The loss of vagal modulation led to significantly increased TNFα levels in tumors and considerably elevated numbers of TAMs. *In vitro* TNFα significantly stimulated growth (*p* < 0.05) and migration (*p* < 0.05) of pancreatic cancer cells. TNFα −/− mice survived significantly longer after tumor implantation (*p* < 0.05), with vagotomy not affecting the prognosis of these animals (*p* > 0.05).

Vagotomy can increase tumor growth and worsen survival in a murine pancreatic cancer model mediated through TAMs and TNFα. Hence, the suppression of TAMs and the modulation of TNFα dependent pathways could offer new perspectives in immunotherapies of pancreatic cancer patients especially with remaining vital tumor cells and lost vagal modulation.

## INTRODUCTION

Pancreatic cancer is the fourth leading cause of cancer death in the western hemisphere. The annual incidence has been steadily rising and it is responsible for approximately 41,000 expected cancer deaths in the United States and 16,000 in Germany [1–5]. The late onset of symptoms, the early development of metastases, and the high morbidities associated with surgery add to its often rapid and fatal course [6]. This is mirrored by the diagnosis usually made in late stage disease [7].

Among the therapeutic options for patients with pancreatic cancer, complete surgical resection is the only chance for cure. Kausch-Whipple pancreaticoduodenectomy and pylorus-preserving pancreaticoduodenectomy are equal in the treatment of pancreatic cancer [8].

These procedures can only be performed in up to 20% of the patients [9, 10]. Even in specialized centers the prognosis for these patients remains poor. This is due to not complete resections in most patients. In up to 76% pancreatic resections show histologically positive resection margins [11, 12] leaving patient with residual tumor load after surgery.

Throughout many operative procedures in the upper gastrointestinal tract the transection of the vagus nerve or its local fibers is unavoidable. Thereby, the anti-inflammatory impact of vagus nerve fibers on tumor infiltrating macrophages and TNFα suppression may get lost. Tracey et al. established the term “cholinergic anti-inflammatory pathway” for this previously unrecognized immunomodulatory circuit that transmits neural inhibition of inflammation [13]. The release of pro-inflammatory cytokines by activated macrophages has a pivotal role in triggering the local inflammatory response and one of the key inflammatory cytokines is TNFα [13]. The active vagus nerve has been demonstrated to suppress TNFα production of macrophages by stimulation of their nicotinic acetylcholine receptors. In addition, recent studies have shown that vagotomy causes increasing TNFα levels in immune cells especially in macrophages and neutrophils [14, 15].

Furthermore, chronic inflammation has been suggested to predispose to cancer formation and progression, and it is very likely that the chemokine system acts as a tumor promoter in this process [16]. In this context members of the TNF superfamily may act as a double-edged sword. Whereas these molecules can induce tumor cell apoptosis through distinct receptors, they can also interact with tumor cells as well as cells of the immune system thereby modulating the tumor's immunological microenvironment. Thus, TNF-related apoptosis inducing ligand (TRAIL) can increase tumor growth in a murine model of pancreatic cancer [17]. Additionally, there are several reports emphasizing the detrimental functions of TNFα in pancreatic cancer [18–20].

In this study we investigated how the destroyed mechanism of neural inhibition following vagotomy affected tumor-growth of pancreatic cancer *in vivo* using a murine syngeneic orthotopic model, thereby shedding light onto the role of TNFα in pancreas cancer.

## RESULTS

### Subdiaphragmatic vagotomy led to significantly increased tumor growth

By visualization of the pancreatic tumor in a small animal 7-Tesla-MRI, we confirmed that vagotomy resulted in increased tumor growth and more invasive tumor morphology. Effectiveness of vagotomy was monitored by increased stomach volume (MRI) and loss of acetylcholine signaling (Supplementary Figure 1).

**Figure 1:**
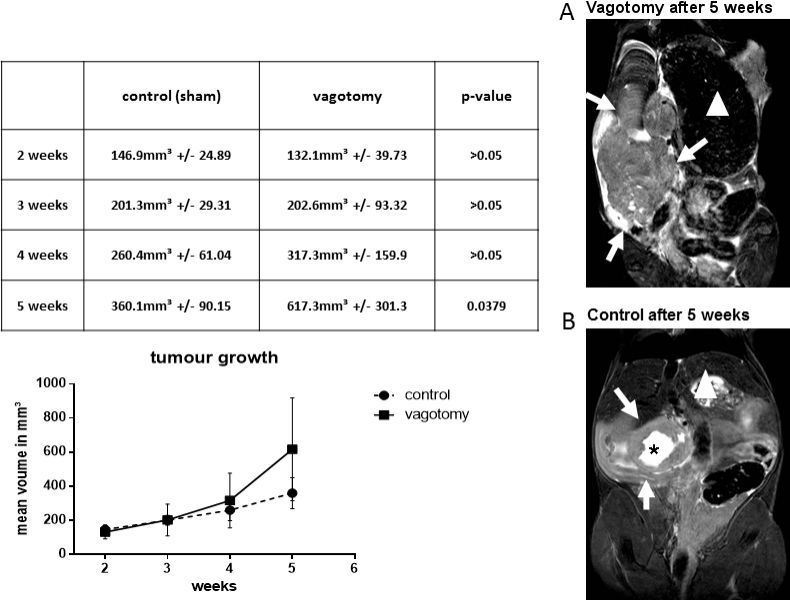
Vagotomy led to significantly increased tumor growth Tumor bearing mice were scanned in a high field 7.0 Tesla MRI scanner for small animals. Vagotomy led to an increased tumor volume 5 weeks after tumor implantation (control (360.1 ± 90.15 mm^3^, *n* = 8) *versus* vagotomy (617.3 ± 301.3 mm^3^, *n* = 7, *p* < 0.05). MRI images (*high resolution T2-TSE images of the coronal plane*) of the vagotomy group show a more irregular disseminated pattern (**A**), whereas MR images of mice with sham-operation resembled a more spherical growth pattern (**B**), often showing a central zone of necrosis (black asterisk). White arrows indicate the tumor. In addition, the large stomach after vagotomy is readily identifiable (stomachs are marked with a white triangle).

Up to the third week after tumor cell implantation there was no significant difference in tumor volumes between the sham and the vagotomy group (Figure 1). But by the end of the third week, the tumor size in mice following vagotomy increased considerably and was significantly larger after 5 weeks compared to sham mice: control: mean 360.1 mm^3^ +/− 90.15 mm^3^, vagotomy: mean 617.3 mm^3^ +/− 301.3 mm^3^, *p* = 0.0379.

**Figure 2:**
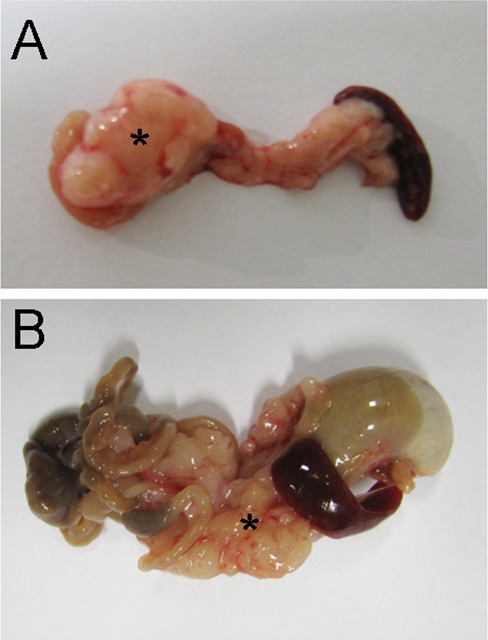
Vagotomy changed tumor morphology Resection specimens of pancreatic carcinoma (5 weeks after orthotopic implantation) in control animals **(A)** and after vagotomy **(B)** After vagotomy the tumor (*) had a more irregular and disseminated morphology compared with the tumor formation after sham surgery.

MRI scans additionally revealed morphological changes in tumor growth. Tumors of mice following vagotomy showed more invasive finger-like MRI images, and displayed a more irregular disseminated pattern (Figure 1A). MRI images of sham-operated mice had a more spherical tumor growth pattern, often showing a central zone of necrosis (Figure 1B). These conspicuous findings could also be confirmed after tumor explantation on the macroscopic pathology (Figure 2A) illustrates a tumor of the sham group and Figure 2B) shows tumor after vagotomy).

### Subdiaphragmatic vagotomy decreased survival

**Figure 3:**
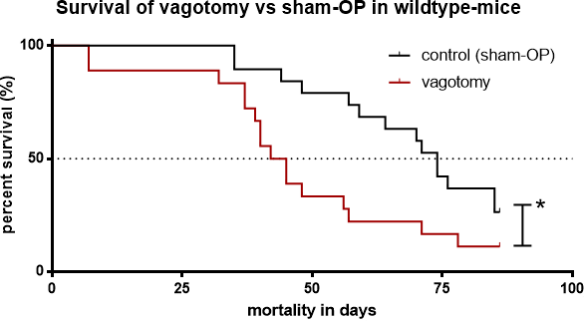
Subdiaphragmatic vagotomy decreased survival Survival of tumor bearing mice with vagotomy (median 44 days) *versus* sham vagotomy (median 74 days, *p* = 0.0172).

The median survival of tumor mice receiving vagotomy was 44 days (*n* = 45, KI 0.3085 to 1.120). In contrast, tumor bearing mice of the sham group without vagotomy displayed a significantly better median survival of 74 days (*n* = 45, KI 0.8928 to 3.241, *p* = 0.0059, Figure 3).

### Vagotomy increased TNFα in pancreatic cancer tissue

**Figure 4:**
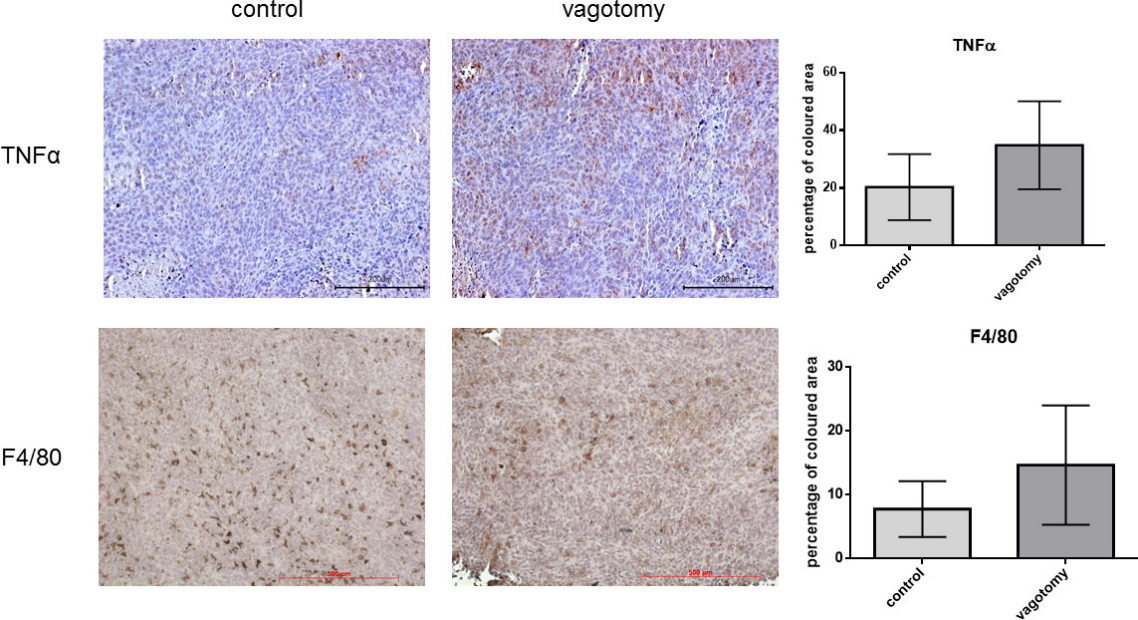
Vagotomy increased TNFα in pancreatic cancer tissue Paraffin sections of explanted pancreatic tumors (5 weeks after orthotopic implantation). TNFα staining shows a significant increase (*p* = 0.0414, *t-test*, one-tailed) of TNFα expression in tumors after vagotomy compared to the control group, whereas the amount of tumor infiltrating F4/80 positive macrophages did not differ significantly (*p* = 0.080, *t-test*, one-tailed).

Immunohistochemistry revealed an increased amount of tumor infiltrating F4/80 positive macrophages following vagotomy. Yet, this was only approaching statistical significance (*p* = 0.089). However, a significant increase (*p* = 0.0414) of TNFα expression was detectable in tumor tissue after vagotomy (Figure 4) compared to the sham group.

### TNFα enhanced proliferation and migration of 6606PDA tumor cells *in vitro*

To determine a direct effect of TNFα on tumor cells, cultures of 6606PDA cells were stimulated by TNFα. Then, proliferation, viability and cell migration of tumor cells were determined.

**Figure 5:**
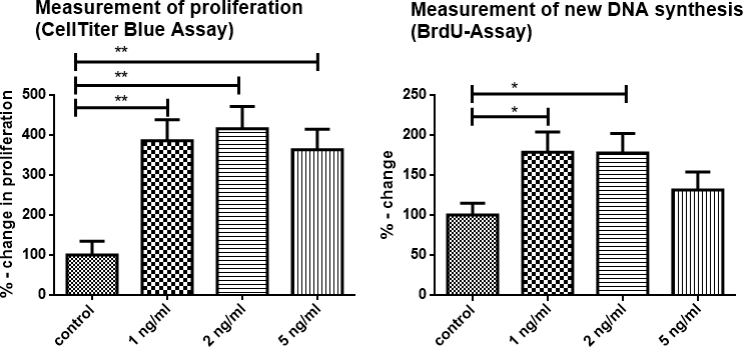
TNFα enhanced proliferation of 6606PDA tumor cells *in vitro*. The effects of TNFα on tumor cell growth (6606PDA cells) were studied *in vitro* using the BrdU-Assay and CTB-Assay. In the CellTiter-Blue assay 1 ng/ml TNFα increased tumor proliferation after 6 hours to a mean of 385.7% of the control, and 2 ng/ml to a mean of 415.7% of the control. 5 ng/ml TNFα did not further increase cell proliferation. BrdU incorporation assay (performed after 24 h of stimulation with TNFα): DNA synthesis was significantly increased with 1 ng/ml TNFα: mean 178.7% of control (+/− 70.94%) *p* = 0.0458 and 2 ng/ml TNFα: mean 177.5% of control (+/− 69.94%) *p* = 0.0174. Stimulation with 5 ng/ml TNFα also resulted in an increase of a mean of 131.4% of control (+/− 63.42%), but did not reach significance (*p* = 0.2786). Representative data are shown from 3 independent experiments.

As detected by a CTB-Assay and a BrdU incorporation assay proliferation and viability of tumor cells were significantly increased following incubation with TNFα (Figure 5). After 6 hours of incubation results of the CTB assay were as follows: 1 ng/ml TNFα: mean 385.7% of control (+/− 139.3%) *p* = 0.0101; 2 ng/ml TNFα: mean 415.7% of control (+/−157.1%) *p* = 0.0109; 5 ng/ml TNFα: mean 363.6% of control (+/−144.3%) *p* = 0.0062.

BrdU incorporation assay, a measure of new DNA synthesis, was performed after 24 h of stimulation with TNFα. DNA synthesis, as a sign of cell proliferation, was significantly increased: 1 ng/ml TNFα: mean 178.7% of control (+/− 70.94%) *p* = 0.0458; 2 ng/ml TNFα: mean 177.5% of control (+/− 69.94%) *p* = 0.0174. 5 ng/ml TNFα: mean 131.4% of control (+/− 63.42%), however this difference was not significant, *p* = 0.2786.

**Figure 6:**
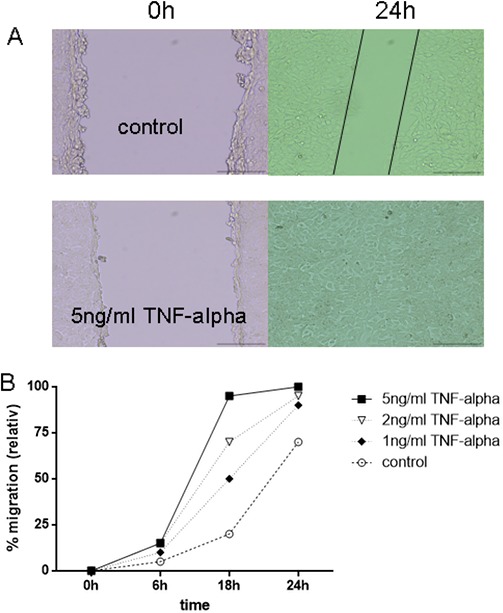
TNFα enhanced migration of 6606PDA tumor cells *in vitro*. Migration of 6606PDA cells was observed using a scratch assay. (**A**) Representative pictures of control vs. 5 ng/ml TNFα: Gap in control after 24 hours is marked with black lines, whereas the gap in stimulated tumor cells is already closed. (**B**) Different concentrations of TNFα resulted in a significant increased migration of the cells into the scratch (*p* < 0.0001).

Migration of 6606PDA cells was observed in an *in vitro* wounding (scratch) assay. Stimulation of pancreatic tumor cells with different concentrations of TNFα resulted in a significant increase of migration of cells into the scratch (*p* < 0.0001) (Figure 6). Stimulation with 1 ng/ml, 2 ng/ml and 5 ng/ml TNFα resulted in a dose dependent stimulation of migration of the cells (Figure 6). After 24 h, cells reached a 65% closure (+/− 5%) of the scratch in the control setting, whereas TNFα caused a closure of the scratch after 24 hours: 1ng/ml TNFα mean 89% (+/− 3.6%), 2ng/ml TNFα mean 92.3% (+/− 2.5%) and 5 ng/ml TNFα 100%.

### TNFα deficiency abolished the effect of subdiaphragmatic vagatomy on survival in pancreatic cancer

**Figure 7:**
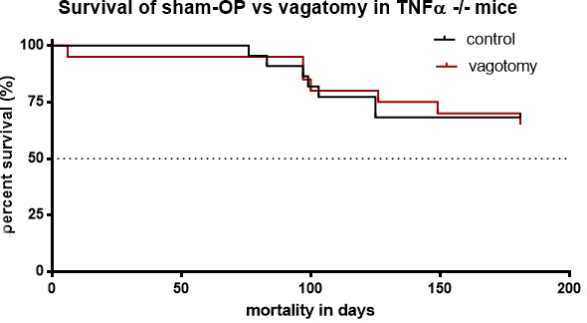
Vagotomy had no significant influence on survival of TNFα deficient mice Survival analyses displayed no significant change of survival in treated (vagotomy, *n* = 21) and untreated (sham-operation, *n* = 22) in TNFα −/− mice, *p* = 0,9027.

To evaluate whether the effect of vagotomy was related to TNFα in pancreatic cancer we analyzed the survival in tumor bearing *TNFα* −/− mice. Comparing vagotomy (*n* = 21*) versus* sham-operation (*n* = 22) in *TNFα* −/− mice we did not find a significant difference in survival, *p* > 0.05 (Figure 7).

Thus, TNFα deficiency abolished the effect of subdiaphragmatic vagotomy on survival in pancreatic cancer indicating that TNFα was a key mediator of vagotomy in this model.

**Figure 8:**
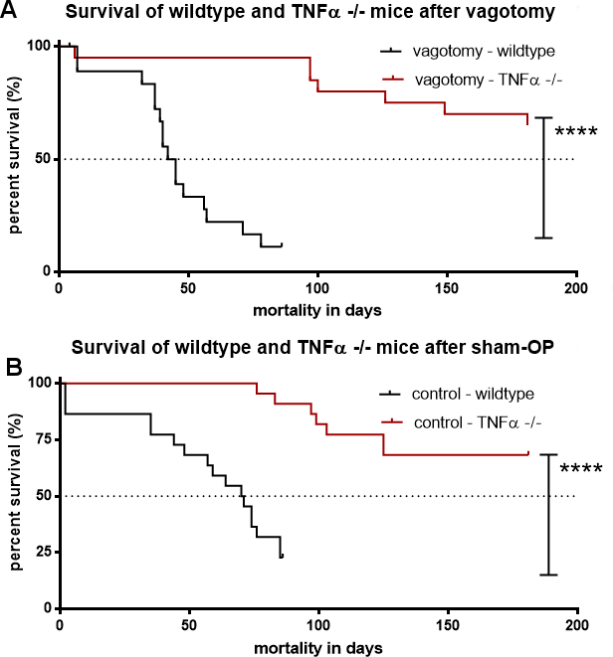
TNFα deficiency abolished the effect of vagotomy on survival in pancreatic cancer (**A**) Survival of *TNFα −/−* and wildtype-mice undergoing vagotomy. Kaplan-Meier survival analyses displayed a significantly shorter survival of the vagotomy-mice with 44 days (KI 0.1292 to 0.9373) in wildtype-mice vs. *TNFα −/−* mice, ****p < 0.0001. (**B**) Survival of TNFα −/− and wildtype-mice undergoing sham-operation. Kaplan-Meier survival analyses displayed a significantly prolonged median survival of sham-op-mice at 74 days (KI 0.3306 to 1.725) in wildtype-mice vs. *TNFα −/−* mice, ****p < 0.0001.

Additionally, TNFα deficiency had a significant benefit on survival in both groups, sham-operation and vagotomy when compared to wild type mice:

The comparison of the median survival of vagotomised mice with 44 days (KI 0.1292 to 0.9373) in wild type mice *versus* the survival of tumor bearing *TNFα* −/− mice indicated the negative effect of TNFα on survival in pancreatic cancer *p* < 0.0001, Figure 8A).

Also, sham operated tumor bearing *TNFα* −/− mice *p* < 0.0001, Figure 8B) lived significantly longer than sham operated wild type mice (median survival of 74 days, KI 0.3306 to 1.725) confirming the importance of TNFα.

## DISCUSSION

The present study showed that the vagus nerve can be one essential piece of the puzzle in tumor control of pancreatic cancer. This effect was strongly dependent on the suppression of TNFα.

In major oncologic surgery of the upper gastro-intestinal tract complete vagus nerve preserving procedures are not feasible. When resecting the pancreatic head or the stomach, preservation of the distal part of the vagus nerve is only rarely possible. Interestingly, local recurrences of these tumor entities are fairly common [21, 22]. Along with the uncommon exception of an R0 resection in pancreatic cancer, the loss of the vagus nerve as an immunomodulator may be a reason for this. In 2000, Tracey et al. described a „parasympathetic immunomodulatory pathway“ [23], in which the vagus nerve played a significant role in the modulation of TNFα release from peritoneal macrophages. Other studies have shown, that transection of the vagus nerve led to inflammation and increased release of TNFα by macrophages [24, 25]. This has been demonstrated for both peritonitis [26] and pancreatitis [27]. In the signal transduction of anti-inflammatory parasympathetic signals, both the nicotinic acetylcholine receptor subtype 7 and the JAK-STAT3 pathway seem to play important roles [28–30]. This study explored the influence of vagotomy on tumor growth and survival in an orthotopic pancreatic cancer model in mice and the possible effects of the parasympathetic immunomodulatory pathway. In an established murine syngeneic orthotopic pancreatic cancer model using pancreatic cancer (6606PDA) cells, sub-diaphragmatic vagotomy *versus* sham surgery was performed.

This study showed that sub-diaphragmatic vagotomy led to enhanced tumor growth and decreased survival in pancreatic cancer. Following vagotomy the suppressing effects of the vagus nerve were lost. This might have triggered TNFα production from tumor associated macrophages (TAM) with a subsequent stimulation of tumor growth. Consequently, we found increased levels of TNFα within the tumor following vagotomy suggesting that the vagal nerve had a direct influence on macrophages within tumor tissues, modulating the local cytokine milieu.

We believe that macrophages were involved in the increase of TNFα since we found consistently elevated numbers of macrophages in wild type mice following vagotomy. Unfortunately, this was only approaching statistical significance. Employing *TNFα −/−* mice we could show that the effects of sub-diaphragmatic vagotomy in pancreas cancer were dependent on TNFα.

Recent studies have also shown that TNFα secreted by macrophages play a pivotal role in cancer progression [31]. In addition, Tracey et al., 2003 [13], could show that macrophages were involved in the rise of TNFα in pancreatitis following vagotomy.

Considering the immunosuppressive role of macrophages we have already shown a tumor growth reduction after macrophage depletion using clodronate [32]. We believe that the inhibition of TNFα could additionally assist chemotherapeutic strategies in pancreatic cancer, possibly by inhibiting immunosuppressive macrophages [31].

The impact of members of the TNF superfamily on tumor progression have been highlighted in several publications. As we had shown before, TNF related apoptosis ligand (TRAIL) could promote tumor growth in murine pancreatic cancer by editing the tumor's immunological microenvironment [17]. The role of TNFα as a promotor of tumor growth in murine pancreatic cancer had been described earlier by Egberts et al., 2008 [33]. Additionally, anti-TNFα-therapy was protective in this setting. The present study could confirm these data using *TNFa−/−* mice. Accordingly, *TNFa−/−* mice were highly protected in pancreatic cancer resulting in a highly significantly increased survival.

TNFα acts through its receptors TNFR1 and TNFR2. TNFR1 is associated with inflammation by activation of the transcription factor NF-κB, JNK and p38-MAPK [34]. More important, TNFR1 activation causes formation of caspase containing complexes (e.g. caspase 8) thereby and via multiple complex pathways including activation of the pro-apoptotic Bcl-2 family proteins and reactive oxygen species inducing apoptosis [35]. TNFR2 mediates anti-inflammatory signaling. It has been shown that TNFR1 is required for T-cell mediated tumor surveillance and tumor rejection. Besides the impact of TNFα on immune cells, there is strong evidence of a direct interaction of TNFα with tumor cells. For human pancreas cancer cell lines, Kalthoff et al. have shown that stimulation with TNFα strongly increased invasiveness with an only moderate anti-proliferative effect. Astonishingly, the stimulation of the murine pancreatic cancer cell line 6606PDA with TNFα led to an enhanced proliferation and migration of this cell line. These data implicate that—besides its interactions with the tumor's microenvironment—TNFα can directly interact with tumor cells thereby promoting proliferation as well as migration. Also, in a murine model using melanoma cells similar results were published by Chopra et al. in 2013 [20]. This has been confirmed for other malignancies like breast cancer [36]. However, Balkwill et al. have already discussed the tumour-suppressing potential of TNFα in 2010 [37]. Therefore, we assume that our data can be applied to pancreatic cancer as well as other malignancies.

Finally, the results of this study could show an influence of the vagus nerve on the local tumor cytokine milieu. The lack of vagal suppression of TAMs led to an increased level of TNFα promotin tumor cell proliferation and migration.

During major pancreatic surgery for cancer disease complete vagus nerve preserving procedures cannot be carried out. However, to improve survival concerning the vagus nerve different therapeutical options may be considered. One option could be the electric stimulation of the vagus nerve as part of a multidisciplinary approach in pancreatic cancer. Also, a drug-based TNFα blockade should be evaluated since Zhao et al., 2016 [38], could recently show that the inhibition of TNFα decreased the cell viability in pancreatic cancer cells. In combination with chemotherapy this prolonged the survival of mice in a murine model of pancreatic cancer.

These results point to a complex role of the nervous system modulating the immune system not only in tumor diseases but possibly also in other immunological pathologies. In combination, these effects may be fatal. In particular, septic complications following major tumor surgery of the upper GI-tract may significantly worsen the prognosis of tumor patients due to the missing anti-inflammatory effects of a transected vagus nerve.

In summary, the combination of frequently occurring residual tumor cells after pancreatic cancer surgery and the loss of postoperative vagal immunomodulation may add to the frequent and early aggressive recurrences of tumors. In this context, the interactions of the vagus nerve, TNFα and the tumor microenvironment have to be further explored in future studies. Especially treatment options focusing on tumor infiltrating macrophages and anti-TNFα-pathways could help to improve the prognosis of pancreatic cancer.

## MATERIALS AND METHODS

### Laboratory animals

Six to eight weeks old male C57BL/6 mice and B6.129S-Tnftm1Gkl/J (TNFα-knock-out) mice with a body weight of 20 to 23 g were obtained from Charles River Laboratories (Sulzfeld, Germany) and allowed to adapt to the new surrounding for seven to fourteen days. They were maintained in an open pathogen-free environment receiving food (ssniff Spezialdiäten GmbH, Soest, Germany) and water ad libitum. Animal rooms had a twelve to twelve-hour light-dark/day-night cycle and were maintained at constant temperature and humidity. Before starting experiments all animal studies had been approved by the ethics committee for animal care of Mecklenburg–Vorpommern, Germany. For survival studies, humane end-points were used according to the Ethics Committee for Animal Care of Mecklenburg-Vorpommern, i.e. animals with any signs of suffering were euthanized/sacrificed using intraperitoneal anaesthesia and finally cervical dislocation. Signs of suffering included but were not limited to obvious changes in behaviour including increasing lethargy; notable changes of fur/ruffled fur; limitation of movements/moving; obvious tumour growth leading to suffering and/or the above signs.

### Cell line and culture

The murine pancreatic adenocarcinoma cell line 6606 PDA was a kind gift from Prof. David Tuveson, Cold Spring Harbor Laboratory, NY, USA. 6606 PDA cells had been isolated from a pancreatic adenocarcinoma in a transgenic C57BL/6 mouse carrying a Kras^G12D^ allele [39]. Cells were maintained in RPMI-1640 medium supplemented with 10% fetal calf serum, 100 U/ml of penicillin and 100 μg/ml of streptomycin (referred to as “complete medium”). Tissue culture reagents were obtained from Gibco (Invitrogen, Carlsbad, California, USA). Cell cultures were kept in a humidified incubator at 37°C with 5% CO_2_. Cell cultures were kept pathogen-free and were regularly tested for *Mycoplasma* species. They were consistently negative for Mycoplasma contamination.

### Orthotopic tumor model

Orthotopic tumor induction was performed as previously described [40]. Briefly, general anesthesia in mice was induced using a combination of ketamine hydrochloride (Ketanest S^®^ Pfizer Pharma, Berlin, Germany) and xylometazoline hydrochloride (Rompun^®^ Bayer HealthCare, Berlin, Germany) at concentrations of 87 mg/kg and 13 mg/kg injected intraperitoneally, respectively. The abdominal cavity was opened by a 1.5 cm wide transverse laparotomy pointing slightly to the right. The head of the pancreas was identified and lifted up by a cotton wool tip. 2.5 × 10^5^ tumor cells in a total volume of 20 μl consisting of equal volumes of PBS and matrigel (Matrigel™ Basement Membrane Matrix, BD Bioscience, San José, CA, USA) were slowly injected into the head of the pancreas. The abdominal cavity was closed by a running single layer 4/0 polyester suture (Catgut, Markneukirchen, Germany). To decrease postsurgical pain and the effects of surgery buprenorphine hydrochloride was subcutaneously injected at a dose of 0.1 mg/kg. All mice were checked daily for their general condition and twice weekly for tumor formation.

### Vagotomy *versus* sham operation

To investigate the consequences of a vagus nerve transection during pancreaticoduodenectomy on tumor growth, either sub-diaphragmatic vagotomy or sham surgery were performed simultaneously in the above described model. By visualizing the stomach in small animal 7-Tesla-MRI (see below), we confirmed that vagotomy had been successful resulting in increased gastric volumes.

Vagotomy was performed as previously described [26]. Briefly, the esophagus was exposed while carefully keeping costal arc, liver, and stomach out of sight. Further preparation was done using a surgical microscope (20×magnification, Leica M651, Bensheim, Germany). The ventral branch of the vagal nerve was exposed and about 3 mm were resected. After its passage through the diaphragm, the esophagus was mobilized on its hepatic side and lifted up. Using diaphanoscopy from the left, the dorsal branch of the vagal nerve could be exposed beneath the esophagus. That branch was then isolated and approximately 3–5 mm were resected. For control purposes, sham operations without transection of the vagal nerve were performed. Following this technique, there were no surgery related fatalities.

### Magnet resonance imaging

Tumor bearing mice were analyzed using magnetic resonance imaging (MRI) in a high field 7.0 Tesla scanner for small animals (Bruker, ClinScan, 7.0 Tesla, 290 mTesla/m gradient strength (Bruker, Ettlingen, Germany) as previously described [40]. For all MRI studies, anesthesia had to be carried out using isoflurane (1%–1.5%). The depth of anesthesia was monitored by the respiratory rate and MRI sequences were triggered by this rate. For all MRI studies, mice were scanned weekly from the second to the fifth week after tumor injection. MRI analyses were performed in a whole mouse body coil (Bruker, Ettlingen, Germany) using a T2-TSE (turbo spin echo) sequence. For the assessment of tumor and gastric sizes we used high resolution T2-weighted images of the coronal as well as the transverse plane (coronal plane: TR (repetition time): ca. 1200 ms; TE (echo time): 41.0 ms; FA (flip angle): 180°; FoV (field of view): 42 mm × 42 mm; matrix: 240 × 320; 24 slices of 0,7 mm per slice, acquisition time: ca. 15 min ; transverse plane: TR: ca. 1250 ms; TE: 41.0 ms; FA: 180°; FoV: 40 mm × 40 mm; matrix: 240 × 320; 24 slices of 0,7mm per slice, acquisition time: ca. 10 min). Images were analyzed employing MIPAV (medical imaging processing and visualization, National Institutes of Health, Bethesda, Maryland, USA). All image slices containing tumors were marked as so called regions of interest (ROI) which facilitated the software calculation of the tumor volume. This analysis was completed with a complex algorithm using all image inherent information including thickness of slices, resolution as well as size of ROIs. The volume of the stomach as a marker for successful vagotomy was calculated in the same manner.

### Immunohistochemistry

The presence of tumor associated macrophages (TAM) and TNFα in primary tumors was analyzed by immunohistochemistry. Explanted tumors were fixed with neutral buffered formalin and embedded in paraffin. Sections (4 μm thick) on glass slides were deparaffinized with xylene and 100% ethanol. Slides were then rinsed and heat induced antigen retrieval was performed using a 10 mM citrate buffer solution adjusted to pH 6.0. After rinsing in phosphate-buffered saline (PBS), endogenous peroxide activity was blocked by incubation in 3% hydrogen peroxide. After washing, non-specific binding was blocked with Aurion-BSA (Aurion, Wageningen, Netherlands) and sections were then incubated with the primary antibody anti-F4/80 (Abd Serotec, Oxford, U.K.) or anti-TNFα (ab 66471, abcam, Cambridge, U.K.) in a humidified chamber overnight at 4°C. F4/80 is a 160 kDa glycoprotein that serving as a well-established mouse macrophage marker. The slides were rinsed in PBS and a biotinylated secondary antibody (anti-rat IgG (Jackson Laboratories, Bar Harbor, Maine USA)) was added. For staining, DAB substrate kits (Vector Laboratories) were used according to the manufacturer's instructions. Negative controls used all reagents except the primary antibody. The intensity of staining was analyzed with Image-J (free software). Two independent observers (D.T. and L.I.P.) examined each slide using a Leica microscope (Leica, Wetzlar, Germany). In addition, the macrophage content was quantified microscopically by counting 3 × 3 fields of view at a magnification of 200 x (total area 250 mm^2^).

### Enzymehistochemistry

The activity of acetylcholinesterase as a functional marker of the vagus nerve was analyzed in cryostat sections of the stomach of vagotomized and control mice using enzyme histochemistry as described by Bruder et al. [41] (data not shown). Briefly, Acetylthiocholine iodide was added to the slides as a substrate for the AChe. Thiocholine was released and lead to the reduction of ferricyanide to ferrocyanide. Ferrocyanide precipitated with copper ions to a brown insoluble copper precipitate.

### *In vitro* wounding (scratch) assay

6606 PDA cells were seeded in triplicates in six-well plates (Becton Dickinson Labware, Bedford, MA). Cells were grown to confluent monolayers and then pre-treated with 1 mM mitomycin (Sigma-Aldrich, St. Louis, USA) for one hour to inhibit cell proliferation. One scratch through the central axis of each plate was gently made using a 200 μl pipette tip. Cells in the 6-well chambers were washed twice with PBS and treated with 0 (control), 1, 2, or 5 mg/ml mouse TNF-α (murine TNFα, T7539, Sigma-Aldrich, St. Louis, USA). Migration of cells into the scratch was observed at four pre-selected time points at 0, 6, 18 and 24 h and photographed at 10-fold magnification. The experiment was repeated three times.

### Cell proliferation and viability assays

Cell viability and proliferation of 6606 PDA cells following stimulation with TNFα were assessed after 6 hours with the CellTiter-Blue Cell Viability Assay (Promega, Mannheim, Germany) and after 24 hours with the BrdU-Assay (Rosche, Mannheim, Germany), according to the manufacturer's protocols. 500 cells *(CellTiter-Blue-Assay)* or 1000 cells (*BrdU-Assay)* per well were seeded in 96-well cell culture plates and treated with 0 (control), 1, 2, or 5 mg/ml mouse TNF-α (murine TNFα, T7539, Sigma-Aldrich, St. Louis, USA) in eight folds. After incubation, fluorescence was measured at 544/590 nm using a FLUOstar Optima plate reader (BMG LABTECH GmbH, Offenburg, Germany) plate reader. Experiments were repeated twice.

### Statistical methods

Statistical analyses of tumor growth and *in vitro* assays were performed using GraphPad Prism (Version 5.01) for Windows software (GraphPad Software, San Diego, CA, USA) using the *T-test*. Results of non-parametric probes were analyzed using the Mann-Whitney-U test. A significance level of 0.05 was applied for all calculations. Statistical differences in survival were assessed using Kaplan-Meier survival procedures and the log-rank test. A *p-value* below 0.05 was considered to be statistically significant.

## SUPPLEMENTARY MATERIALS FIGURES AND TABLES


