# Supplementary material for: Subdiaphragmatic vagotomy promotes tumor growth and reduces survival via TNFα in a murine pancreatic cancer model
Source: Oncotarget. 2017 Feb 2;8(14):22501–12. doi: 10.18632/oncotarget.15019 (PMC5410240; doi:10.18632/oncotarget.15019)
Supplement: Supplementary file 1 [file oncotarget-08-22501-s001.pdf]

Subdiaphragmatic vagotomy promotes tumor growth and reduces survival via TNFα in a murine pancreatic cancer model

Supplementary Materials

Effectiveness of subdiaphragmatic vagotomy in the murine pancreatic cancer model

Employing small animal 7-Tesla-MRI, we confirmed that vagotomy resulted in increased gastric volumes. Supplementary Figure 1A, 1B) shows the regular gastric volume with 295.2 ± 86.08 mm^3 in control animals ($n = 7$) at two weeks and 304.7 ± 59.96 mm^3 at five weeks after tumour implantation. Following vagotomy ($n = 10$), the volume was increased to 1330.0 ± 286.5 mm^3 two weeks after tumour implantation and 1521.0 ± 248.1 mm^3 five weeks after tumour implantation. These differences were highly significant ($p < 0.0001$).

As a functional control confirming successful vagotomy, the activity of acetylcholinesterase (AcE) in the stomach wall was measured by immunohistochemistry. Supplementary Figure 1C illustrates the much higher level of AcE in the stomach of control vs. vagotomised mice five weeks post-operatively. In addition, a much thicker stomach wall was seen after vagotomy.

Despite the obvious dysfunction of the gastric emptying after vagotomy, there was no significant difference in changes of body weights in the time elapsed ($p = 0.9037$, data not shown).

Both groups (sham and vagotomy) presented with a characteristic loss of weight in the first week after operation (–6% after sham-vagotomy and –8% after vagotomy). In the following weeks, both groups gained weight considerably (+5% after sham-vagotomy and +7% following vagotomy). Due to an increasing tumour burden, both groups lost body weight after the second week.

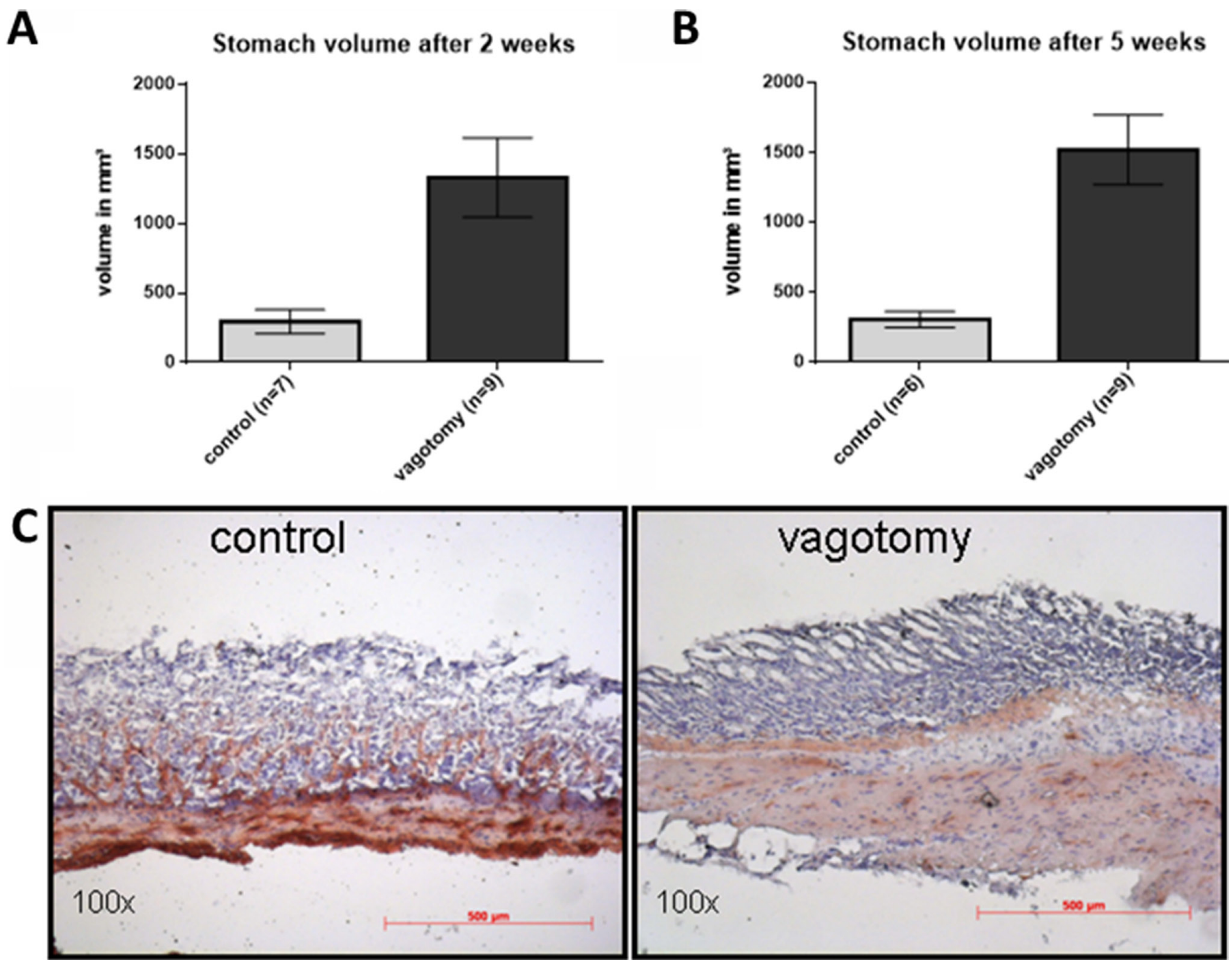

Supplementary Figure 1: Vagotomy is effective and increased stomach volume. Stomach volume: Stomach volume in tumor bearing mice was measured by MRI 2 weeks (**A**) and 5 weeks (**B**) after vagotomy (2 weeks: 1330.0 mm^3 ± 286.5 mm^3 and 5 weeks: 1521.0mm3 ± 248.1mm3) and sham-surgery (control) (2 weeks: 295.2mm^3 ± 86.08 mm3 and 5 weeks: 304.7 mm^3 ± 60.0 mm^3). Vagotomy significantly increased the organ volume ($p < 0.001$) at both time points compared to control. Pancreatic carcinoma did not lead to a delayed gastric emptying (control 2 weeks vs. control 5 weeks, $p = 0.8$). Enzyme histochemistry: Figure (**C**) illustrates the much higher amount of Acetylcholinesterase in the stomach and a much thicker stomach wall of control vs. vagotomy mice.
